# RELATIONSHIP QUALITY PATTERNS AND DISABILITY IN LATER LIFE

**DOI:** 10.1093/geroni/igae098.1528

**Published:** 2024-12-31

**Authors:** Madison Sauerteig-Rolston, Kenneth Ferraro

**Affiliations:** Indiana University School of Medicine, Indianapolis, Indiana, United States; Purdue University, West Lafayette, Indiana, United States

## Abstract

Informed by the life course perspective and stress process model, this study examined the gendered relationship between patterns of relationship quality (identified as high quality, adverse, indifferent, or ambivalent) and the onset of disability in later life. Cross-sectional and longitudinal models were estimated using nationally representative data from the Health and Retirement Study. Logistic regression models were used to examine cross-sectional associations between relationship quality and wave 1 (W1) disability (i.e., occurrence). Weibull accelerated failure-time models were employed to examine the association between relationship quality and age of onset of disability 12-14 years later (i.e., incidence). We tested for moderation between relationship quality and disability by gender. At W1, older adults in adverse relationships had higher odds of disability than older adults in high quality relationships (OR=1.26, 95% CI=1.11-1.44). In addition, men who reported adverse or indifferent relationships had a higher probability of disability than women in adverse or indifferent relationships (respectively, 2nd differences= 0.029, 0.032). Over time, early onset of disability was associated with adverse (β = -0.07, p < 0.001) and ambivalent relationships (β = -0.07, p < 0.001). It is important to consider the complex and multidimensional aspects of social relationships to understand how relationships influence later-life disability. Not only are adverse relationships associated with an earlier onset of disability in later life, but ambivalent relationships, those identified by high levels of support and high levels of strain, are detrimental for physical functioning among older adults.

